# Structure and Assembly of Group B Streptococcus Pilus 2b Backbone Protein

**DOI:** 10.1371/journal.pone.0125875

**Published:** 2015-05-05

**Authors:** Roberta Cozzi, Enrico Malito, Maddalena Lazzarin, Annalisa Nuccitelli, Andrea Castagnetti, Matthew J. Bottomley, Immaculada Margarit, Domenico Maione, C. Daniela Rinaudo

**Affiliations:** Novartis Vaccines and Diagnostics, Siena, Italy

## Abstract

Group B *Streptococcus* (GBS) is a major cause of invasive disease in infants. Like other Gram-positive bacteria, GBS uses a sortase C-catalyzed transpeptidation mechanism to generate cell surface pili from backbone and ancillary pilin precursor substrates. The three pilus types identified in GBS contain structural subunits that are highly immunogenic and are promising candidates for the development of a broadly-protective vaccine. Here we report the X-ray crystal structure of the backbone protein of pilus 2b (BP-2b) at 1.06Å resolution. The structure reveals a classical IgG-like fold typical of the pilin subunits of other Gram-positive bacteria. The crystallized portion of the protein (residues 185-468) encompasses domains D2 and D3 that together confer high stability to the protein due to the presence of an internal isopeptide bond within each domain. The D2+D3 region, lacking the N-terminal D1 domain, was as potent as the entire protein in conferring protection against GBS challenge in a well-established mouse model. By site-directed mutagenesis and complementation studies in GBS knock-out strains we identified the residues and motives essential for assembly of the BP-2b monomers into high-molecular weight complexes, thus providing new insights into pilus 2b polymerization.

## Introduction

*Streptococcus agalactiae* (also known as Group B *Streptococcus* or GBS) is an opportunistic Gram-positive pathogen causing early and late onset neonatal invasive diseases including sepsis, pneumonia, and meningitis, as well as severe infections in the elderly and in immune-compromised patients [1, 2]. Infections in newborns are mainly acquired during delivery by direct mother-to-baby transmission of the pathogen, which is the primary colonizer of the ano-genital mucosa of healthy women [3].

The identification in streptococcal pathogens of pili, which are long proteic appendages extending from the bacterial cell surface have aroused great interest because of their possible role in virulence and protective capacity against infection [4]. GBS expresses three structurally distinct pilus types, each containing at least two antigens capable of eliciting protective immunity in mouse models, confirming a key role in bacterial pathogenesis [5–8]. Remarkably, extensive analysis of pilus distribution and conservation in multiple collections of isolates from different hosts (human and bovine) and geographic areas indicated that all GBS strains carried at least one or a combination of two pilus islands [6, 9].

The three GBS pilus types are coded by distinct genomic loci containing features typical of Gram-positive pilus islands (PIs). These islands, named PI-1, PI-2a and PI-2b, encode three structural pilus proteins, corresponding to the major pilus subunit (backbone protein, BP) that forms the pilus shaft and two ancillary proteins that appear to be located at the pilus tip (AP1) and at the base (AP2) as anchor protein of the pilus to the bacterial cell-wall. Additional genes present in the pilus clusters code for class C sortase enzymes that catalyze pilin protein polymerization and are essential for pilus assembly. PI-2a and PI-2b represent two variants of Pilus Island 2 since they are alternatively present in the same genomic locus [7, 10, 11].

Pilus Island 2b (PI-2b) has been commonly found in the epidemiologically relevant GBS serotype III-ST-17 lineage [9]. The sequence type ST-17 defines a highly virulent serotype III clone strongly associated with a deadly form of the infection called late-onset disease (LOD), which is characterized by meningitis in infants after the first week of life [12, 13]. Genomic studies showed that the ST-17 hypervirulent clone is a homogeneous group of strains that displays a conserved combination of secreted/surface proteins, including the pilus type 2b [14]. Prior to the discovery of pilus-structures in Gram-positive bacteria, the backbone protein of pilus 2b (BP-2b) was described as a GBS surface invasin (named surface protein of GBS 1, Spb1), due to its ability to promote invasion of epithelial cells [15]. Indeed, Spb1 was identified during a search for virulence factors unique to this highly virulent clonal lineage by subtractive hybridization experiments, suggesting a key role in GBS pathogenesis [15].

The current model proposed for pilus assembly in Gram-positive bacteria is based on a transpeptidation mechanism from backbone and ancillary pilin precursor substrates catalyzed by class C sortases [16], except for pili in *S*. *pyogenes* where a class B sortase (Spy0129) is known to catalyze pilin protein polymerization [17]. These enzymes recognize specific sequence elements and/or residues in the pilin subunit that are essential for pilus assembly, and well conserved among pilin-subunits in different bacteria. The three main motives include (i) the pilin motif (consensus WxxxVxVYPKN), wherein the lysine residue (K) participates in sortase catalyzed amide bond formation by reaction with the C terminus of the next subunit molecule during polymerization; (ii) a cell wall sorting signal (CWSS) containing the sortase recognition site LPxTG motif, typical of cell wall-anchored proteins, and (iii) the E-box motif (consensus YxLxETxAPxGY), important for the proper folding of the pilin proteins and subsequently necessary for the pilus polymerization [18–20]. While the assembly of the well characterized GBS pili 1 and 2a occur following the classical model [7, 10, 11], the pilus 2b seems to differ slightly: the pilus 2b components do not contain the canonical conserved primary sequence of the pilin motif described for pilus polymerization in Gram-positive bacteria, making difficult the identification of the key residues involved in pilus assembly.

In recent years, the X-ray crystal structures of several Gram-positive pilin proteins from *Corynnebacteium diphtheriae* [21, 22], *Actinomyces species* [23, 24], *Streptococcus pyogenes* [25], *Streptococcus pneumoniae* [26–30], *Streptococcus agalactiae* [8, 31–33] and *Bacillus cereus* [34] have been described. Despite low sequences similarities, these proteins share a very similar IgG-like fold domain organization. Each domain is stabilized by an intramolecular isopeptide bond commonly formed by Lys-Asn residues (although Lys-Asp bonds also exist [35, 36]), located in a largely hydrophobic pocket comprising several aromatic residues, including a bond-catalyzing aspartyl or glutamyl residue. Intriguingly, in the backbone protein of GBS pilus 2a (BP-2a) and in other major pilins this glutamate is the same conserved residue present in the E-box motif [20].

Here we report the structural and functional characterization of the backbone protein of pilus 2b (BP-2b). We solved the X-ray structure of a fragment of the protein (lacking the N-terminal first 184 residues) encompassing domains D2 and D3. Both domains revealed an IgG-like fold organization, typical of the pilin subunits, and the presence of internal isopeptide bonds. Using the three-dimensional information we generated the single structurally well-defined domains as recombinant proteins and compared their protective immunity with the full length protein in a well-established mouse model. Finally, by site-directed mutagenesis and complementation studies in GBS strains we identified the non-canonical residues/motives essential for the BP-2b protein polymerization.

## Materials and Methods

### Bacterial Strains, Media, and Growth Conditions

The *S*. *agalactiae* strains were grown at 37°C in 5% CO_2_ in Todd Hewitt Broth (Difco Laboratories) or in trypticase soy agar supplemented with 5% sheep blood. GBS strains A909 and COH1 were kindly provided by Dr. Dennis Kasper (Harvard Medical School, Boston, MA, USA). GBS strain ABC020017623 carrying only the pilus island 2b (PI-2b) was obtained from the Center for Disease Control and Prevention (CDC).

### Cloning, expression and purification of recombinant proteins

Genomic DNA was isolated from GBS strains by a standard protocol for Gram-Positive bacteria, by mutanolysin-treatment of bacterial cells using a GeneElute Bacterial Genomic DNA kit (Sigma-Aldrich) according to the manufacturer’s instructions.

Gene fragments (locus tag SAK_1440), which encodes the wild type BP-2b protein (GenBank accession number: ABA44859.1, UniProt code Q3K0A5) were PCR-amplified from the GBS strain A909 genome (NCBI Reference Sequence: NC_007432.1). Expression constructs encoding BP-2b_30-468_ without the predicted N-terminal signal peptide and the C-terminal transmembrane domain, and for BP-2b_185-468_ (BP-2b_D2+D3_), lacking the N-terminal domain 1, and the three single domains (D1_30-184_, D2_185-356_, D3_357-468_) were cloned into the pET21b+ vector and expressed as C-terminal His-tagged proteins. The recombinant mutant BP-2b_D2+D3(E423A)_ was generated by site-directed mutagenesis using as template the wild type genes and the KAPA Hi-FI polymerase (KAPA Biosystem). Proteins were expressed in *E*. *coli* BL21 (DE3) T1R (Novagen) cells grown in LB, or in Biosilta Enbase media. The soluble proteins were chemically extracted by using the CelLytic reagent (Sigma-Aldrich) solubilized in Tris pH 7.5, 300mM NaCl, 10mM imidazole, followed by centrifugation to remove cell debris. The BP-2b proteins were purified from the supernatant using an FF-Crude His-Trap HP nickel chelating column (Amersham Bioscience). The recombinant proteins, eluted with 300mM imidazole, were concentrated by ultrafiltration to 10 mg/ml and loaded onto HiLoad 26/60 Superdex 75 (Amersham Biosciences) equilibrated in 50mM Tris-HCl (pH 7.5), 75 mM NaCl. The protein abundance in pure fractions was quantified with the bicinchoninic assay (BCA, Pierce).

### Crystallization, data collection and structure determination

Crystallization experiments were carried out using the sitting drop vapour diffusion method in 96-well low profile Intelliplate crystallization plates, using a Crystal Gryphon (Art Robbins) robot. Equal volumes (200 nL) of the rBP-2b_D2+D3_ protein concentrated to 90 mg/ml, and crystallization buffers were mixed and incubated at 21°C, and crystals grew in a solution containing 0.2 M calcium acetate and 20% (w/v) polyethylene glycol 3,350 in approximately three weeks (S1A Fig). All crystals were mounted in cryoloops using 10% Ethylene glycol and 10% glycerol as cryoprotectant, prior to cooling to 100 K for data collection.

X-ray diffraction data were collected on beamline BM30A of European Synchrotron Radiation Facility (ESRF), Grenoble, France. All diffraction data were processed with iMosflm [37], scaled with Aimless [38] and crystallographic manipulations were carried out with the CCP4 package [39]. The crystals belonged to space group *P*2_1_, with unit cell dimensions a = 47.6, b = 53.9, c = 54.7, and β = 90.9, and contained 1 molecule in the asymmetric unit (Matthews coefficient 2.08 Å^3^ Da^-1^, for a solvent content of 41%). The structure of BP-2b_D2+D3_ was solved at 1.06 Å resolution using the automated molecular-replacement pipeline Balbes [40], using as initial input coordinate templates multiple domain models from PDBs 3ich, 3qdh, and 2h9g, which correspond to structures of cyclophilin B, fimbral adhesin FimA, and precursor 10B of human tumor necrosis factor receptor, respectively. The models were automatically selected by Balbes among 21 structures found to share above 15% sequence identity with the sequence of BP-2b, while the best sequence identity found (~39%) was with the fimbral adhesin FimA (pdb 3qdh). Initial refinement of the solution found by Bables with Refmac [38] resulted in R_work_/R_free_ 51.1/52.8, and a Q factor of 45.7% [40], while subsequent refinement in Phenix [41] and rebuilding in Coot [42] gave final R_work_/R_free_ 12.6/14.2. The final refined model includes residues 185–468, while regions that were present in the crystallized construct but were not included in the final refined model because of insufficient electron density include residues 287–295 and the C-terminal His-tag residues present in the construct (EHHHHHH). In addition, three calcium ions and two ethylene glycol molecules, deriving from the crystallization medium and the cryoprotectant solution, respectively, were modelled in the final coordinates that were deposited in the protein data bank (PDB) with code 4uzg. Molecular graphics images were generated with Pymol (http://www.pymol.org). Data collection and refinement statistics are shown in Table 1.

**Table 1:** Data collection and refinement statistics.

***Data collection***	
Space group	*P* 2_1_
Cell dimensions	
*a*, *b*, *c* (Å)	47.6, 53.9, 54.7
α, β, γ (°)	90, 90.9, 90
Resolution (Å)	47–1.06
*R*_sym_ or *R*_merge_ * (%)	3.4 (26.2)
*I*/σ*I* *	17 (2.9)
Wilson B-factor	7.9
Completeness (%)*	95.2 (88)
Redundancy*	2.6 (1.8)
***Refinement***	
Resolution (Å)	47–1.06
No. reflections	119340
*R*_work_/*R*_free_ (%)	12.6/14.2
No. atoms	
Protein	2156
Ligand/ion	8 (2 ethylene glycol)/3 (calcium)
Water	494
B-factors (Å^2^)	
Protein	11.7
Ligand/ion	10.7
Water	25.8
R.m.s deviations	
Bond lengths (Å)	0.01
Bond angles (°)	1.42
Molprobity	
Ramachandran favored	99
Ramachandran outliers	0
Clashscore	4.65

*Highest resolution shell is shown in parenthesis.

*R_sym_ = Σ_hkl_ Σ_i_ |I_i_(hkl) - <I(hkl)>| / Σ_hkl_ Σ_i_ I_i_(hkl)*

*R_work_ = Σ||F_(obs)_|- |F_(calc)_||/Σ|F_(obs)_|*

*R_free_ = as for Rwork, but calculated for 5.0% of the total reflections that were chosen at random and omitted from refinement.*

### Differential scanning fluorimetry (DSF)

The thermostability of the recombinant protein constructs of BP-2b used in this study was assessed with differential scanning fluorimetry (DSF). Each purified protein was diluted in DSF buffer (25 mM Tris pH 8, 150 mM NaCl) to a final concentration 20 μM. 4 μl of 10× solution of Sypro Orange freshly prepared from a 5000× stock (Invitrogen) were added to each protein solution. The final volume of the reaction mixture was 40 μl in a 96-well plate (Thermo Fast 96-ABgene). The plate also included a baseline control containing Sypro Orange with DSF buffer only. The plate was sealed with optical tape and was centrifuged to remove any bubbles. The melting point (T_m_) of each protein was determined by ramping from 25°C to 100°C with a scan-rate increment of 1°C per min increase, taking a fluorescence measurement for each 1°C step. The unfolding profile and the melting temperature were monitored by a quantitative PCR thermo cycler (Stratagene) as reported previously [43]. All DSF experiments were repeated at least 3 times. Fluorescence intensities were plotted as a function of temperature and the reported T_m_ is the inflection point of the sigmoid curve determined using GraphPad Prism 5.0 software.

To investigate the thermal stability of BP-2b in presence of calcium ions, the BP-2b protein solution was first mixed with 150 mM EDTA and then incubated at room temperature for 30 minutes. Then EDTA was removed by a buffer exchange step (PD10 column, GE Healthcare), and different CaCl_2_ concentrations (from 0 to 10 mM) were added to the protein solution, followed by DSF experiments performed as described above.

### Limited proteolysis assay

Sequencing grade Trypsin (Promega) was dissolved in the buffer provided to a final concentration of 0.2 μg/μl and was activated by heating for 10 minutes at 37°C. 2 μg of trypsin and 200 μg of the recombinant constructs of BP-2b were mixed in a final volume of 100 μl in buffer 25 mM Tris-HCl, 100 mM NaCl pH 7.5. The reactions were incubated at 37°C. Samples were collected after 5, 10, 20 and 30 minutes, the proteolysis was then quenched by adding SDS Sample buffer, boiled for 5 minutes and analyzed by SDS-PAGE. Proteins were transferred to PVDF membrane by electroblotting, stained with Coomasie blue and proteolytically resistant species were identified by N-terminal sequencing at PRIMM SRL.

### Construction of GBS complementation vectors and site-directed mutagenesis

The in-frame deletion mutant strain for the backbone protein of pilus 2b (ΔBP-2b) was generated using Splicing by Overlap Extension (SOE) PCR as described previously [44]. For the generation of the complementation vector pAM_BP-2b, a DNA fragment corresponding to *BP-2b* gene (locus tag SAK_1440) was PCR amplified from GBS A909 genome, and the product was cloned into the E. *coli*-streptococcal shuttle vector pAM401/gbs80P+T, containing the promoter and terminator regions of the *gbs80* gene (SAG_0645) previously described [7]. The complementation vectors expressing mutated forms of BP-2b (pAM-BP_K77A_, pAM-BP_K82A_, pAM-BP_K118A_, pAM-BP_K175A_, pAM-BP_E423A_ and pAM-BP_ΔLPXTG_ were generated by site-directed mutagenesis, using the polymerase incomplete primer extension (PIPE) method [45] performed using the pAM_BP-2b vector as template for the introduction of specific mutations. Replacement or deletions of selected amino acids was performed individually with synthetic oligonucleotide primers. Mutations were verified by DNA sequencing. All complementation vectors were electroporated into the GBS knockout strain Δ*BP-2b* and complementation was confirmed by checking protein expression by Western blot analysis.

### Western blot analysis

Mid-exponential-phase GBS cells were resuspended in 50 mM Tris-HCl pH 6.8 containing 400U of mutanolysin (Sigma-Aldrich, St. Louis, MO, USA) and complete protease inhibitors (Roche, Indianapolis, IN, USA). The mixtures were then incubated at 37°C for 2 hours and cells lysed by 3 cycles of freeze/thawing. Cellular debris was removed by centrifugation. Total protein extracts were quantified by a BCA protein assay and equal amounts of proteins were resolved on 3–8% NuPAGE precast gels (Invitrogen, Carlsbad, CA, USA) by sodium dodecyl sulfate—polyacrylamide gel electrophoresis (SDS-PAGE) and transferred to nitrocellulose. Transfer efficiency of proteins on the membrane and that an equal amount of protein sample was loaded in each well were checked by Ponceau staining. Membranes were probed with mouse antiserum directed against the pilus 2b backbone protein (α-BP-2b, 1:1000 dilution), followed by a rabbit anti-mouse horseradish peroxidase-conjugated secondary antibody. Bands were then visualized using an Opti-4CN substrate kit (Bio-Rad, Hercules, CA, USA). The antiserum specific for the pilus 2b backbone protein (anti-BP-2b) was produced by immunizing CD1 mice with the purified recombinant protein BP-2b.

### Ethics Statement

All animal studies were carried out in compliance with current Italian legislation on the care and use of animals in experimentation (Legislative Decree 116/92) and with the Novartis Animal Welfare Policy and Standards. Protocols were approved by the Italian Ministry of Health (authorization 21/2009-B) and by the local Novartis Animal Ethical Committee (authorization AEC 200825). Following infection, mice were daily clinically monitored to check their ability to feed, reactivity and motility, and cutaneous redness. Sick pups, with dark red skin, empty stomachs (visualized as not white as in healthy pups), weak and unresponsive when handled, were euthanized by decapitation in agreement with Novartis Animal Welfare Policies. No unintended deaths of animals occurred during this study.

### Mouse active maternal immunization model

A mouse maternal immunization/pup challenge model of GBS infection was used to verify the protective efficacy of the antigens, as previously described [5]. In brief, groups 8 CD-1 female mice (6–8 weeks old) were immunized on days 1, 21 and 35 with either buffer (PBS) or 20 μg of protein per dose in aluminium hydroxide formulations. Mice were then mated, and their offspring were challenged intraperitoneally, within 48 h of birth, with a GBS (COH1 strain) dose calculated to induce dead in 90% of the pups. Protection values were calculated as [(% dead in control—% dead in vaccine)/% dead in control] x100. Statistical analysis was performed using Fisher’s exact test.

## Results

### Structure determination of the protease-resistant fragment of BP-2b pilus subunit

The backbone protein of GBS Pilus Island 2b (BP-2b) consists of 502 residues and carries a C-terminal cell wall sorting signal (CWSS) with the sortase recognition site LPSTG encompassing residues 468–473. The protein shows low sequence identity ranging from 12 to 16% (calculated on the entire sequence) with other known backbone pilin subunits from either GBS or other Gram-Positive bacteria. In order to elucidate its three-dimensional structure we attempted to crystallize the recombinant BP-2b protein for structural studies. The full-length ectodomain, and shorter constructs, of BP-2b were produced in *E*. *coli* and purified via a C-terminal 6-His-tag using standard chromatographic techniques (see Experimental Procedures), yielding approximately 10mg of purified protein from 2g of wet biomass.

Initially, crystallization trials were performed using the full-length protein (residues 30–468, BP-2b_30-468_), lacking only the N-terminal signal peptide and the C-terminal sorting motif. All attempts to obtain crystals with this full-length protein failed, while crystals were obtained for the protein-fragment containing residues 185–468 (rBP-2b_185-468_) (S1A Fig). The successfully crystallized BP-2b_185-468_ construct was designed on the basis of the N-terminal sequencing of the recombinant BP-2b protease-resistant fragment formed after storage of the full length BP-2b at 4°C for approximately six months (S1B Fig). SDS-PAGE analysis confirmed the high purity of the BP-2b_185-468_ construct after immobilized-metal affinity chromatography (IMAC) and size-exclusion chromatography (SEC) purification steps (S1C Fig). Analytical SEC revealed that the BP-2b fragment is monomeric in solution, the theoretical molecular weight is 32kDa, compatible with the apparent molecular weight calculated by SEC, where the BP-2b_185-468_ protein fragment eluted as a single peak after Ovalbumin (44 kDa) and prior to Myoglobin (17 kDa) MW standards. No peaks indicative of dimers, or oligomers were detected (S1D Fig).

### Overall Structure

The crystal structure of BP-2b_185-468_ was solved and refined at 1.06 Å resolution by molecular replacement using *Balbes* [40]. A search of the Protein Data Bank (PDB) using the sequence of BP-2b_185-468_ revealed only low overall sequence identity (24%) with the adhesin FimA from *Actinomyces fimbriae*, the structure of which was reported previously (PDB 3qdh) [23]. However, the sequence identity increased to ~39% when considering a subset of only 88 common residues, this being the fragment with highest similarity among a total of 21 structures found in the PDB, all sharing sequence identities above 15% with BP-2b_185-468_. Consequently, a template model prepared using coordinates of chain A of FimA (3qdh) was used for molecular replacement in *molrep* [46], and this yielded a solution with electron density maps of excellent quality that allowed straightforward model building and refinement of the BP-2b_185-468_ structure.

**Fig 1:**
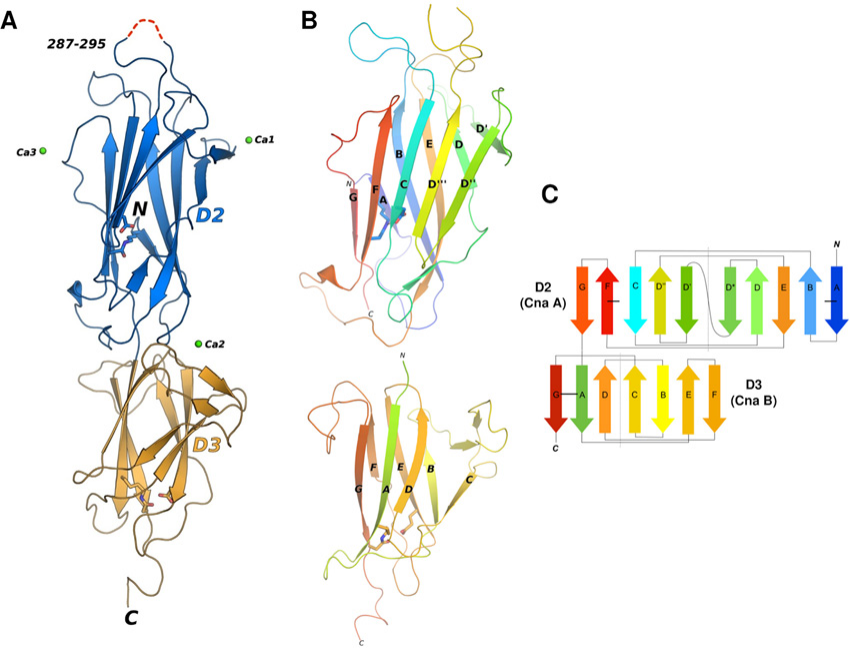
Overall structure and topology of the backbone pilus protein BP-2b. **(A)** The structure of BP-2b is depicted with light blue and orange cartoons for domains D2 and D3. Intradomain isopeptide bonds in D2 and D3 are shown with sticks, while green spheres show calcium ions located on the surface. The red dashed lines indicate the only residues that could not be modelled due to the lack of electron density. (**B)** Cartoon representation of D2 and D3 of BP-2b, with the two domains reoriented with respect to the view in A), and colored with a gradient according to the topology diagram on the right. (**C**) Topology diagram for D2 and D3 of BP-2b, with their core β-strands labeled A—G in rainbow style colored as in panel B. The strands linked by isopeptide bonds in D2 and D3 are marked by thick horizontal black lines.

The asymmetric unit of the BP-2b_185-468_ crystals contains a single molecule, composed of two distinct domains, each adopting a modified IgG-fold. We named the two domains as D2 (residues 185–351) and D3 (residues 352–468) (Fig 1A) and the N-terminal protease-sensitive region (residues 30–183), which is missing in the crystallized fragment, as domain D1. Residues 287–295 of D2 could not be modeled due to lack of electron density, suggesting disorder or flexibility of this region that may interact with the D1 domain in the full length BP-2b protein (Fig 1A).

**Fig 2:**
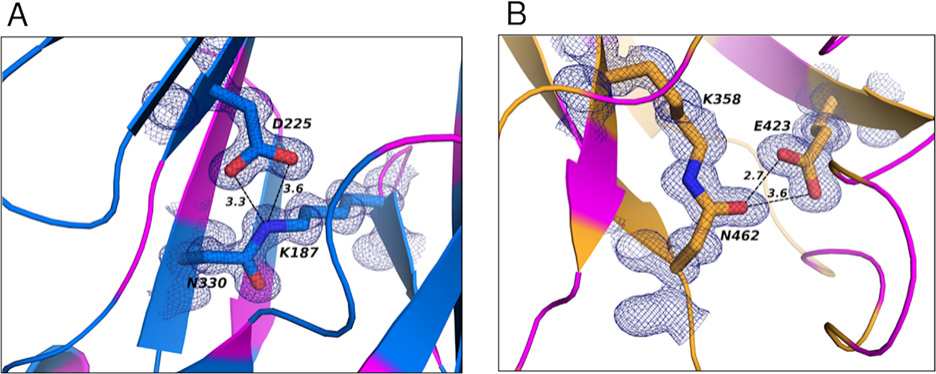
Isopeptide bonds of BP-2b. Domains D2 and D3 are colored as in Fig 1 in blue and orange, respectively. Isopeptide bonds between Asn330 and Lys 187 for D2, and between Asn462 and Lys358 in D3 are shown with blue and orange sticks, and 1σ 2*F*o-*F*c electron density map around this region is shown as blue mesh (carve = 1.1). The magenta colored regions in (A) and (B) show the location of hydrophobic residues surrounding the isopeptide bonds. Hydrogen-bonds between the isopeptide bonds and the nearby Asp (225, D2) and Glu (423, D3) are shown with black dashed lines.

The D2 domain exhibits the typical CnaA-type fold [47], in which ten strands form a partially open β-barrel (Fig 1B). The β-barrel is composed of two parallel β-sheets; each containing five anti-parallel strands (Fig 1C). These two β-sheets are connected by an inter-sheet isopeptide bond between residues Lys187 and Asn330 belonging to strands A and F, respectively (Figs 1A, 1C, and 2A). The D3 domain exhibits a seven stranded CnaB type fold, with reverse-Ig topology, first identified in collagen-binding *S*. *aureus* Cna B-region repeats and in the N1 domain of GBS52 [32], the minor pilin of GBS PI-1. As such, the overall β-sandwich of D3 is made of three- and four-stranded sheets (Fig 1B and 1C). The first (A) and last (G) strands of D3 are connected by an intra-sheet isopeptide bond involving residues Lys358 and Asn462 (Figs 1C and 2B). Three connecting loops in D3 interact with two loops of D2, thus stabilizing the interface between the two domains (Fig 1A).

Strong peaks of positive difference electron densities were observed on the surface of BP-2b, suggesting binding of metal ions to both D2 and D3 (Fig 1A). Based on the observation of nearby residues and water molecules, and based on the inclusion of 0.2M calcium chloride in the crystallization medium, these peaks were modeled as calcium cations. One of the calcium ions (Ca2) is located approximately at the interface between D2 and D3 and interacts with D3 residue Val390 (through its carbonyl backbone), and the side chain oxygen atoms of residues Glu391 and Asn389 (S2 Fig). In addition, four water molecules are also located nearby and make favorable interactions with the calcium ion, for a total of seven oxygen atoms donors and a pentagonal bipyramidal binding geometry. The other two calcium ions, Ca1 and Ca3, are also coordinated by seven oxygen atoms each, mostly derived from the side chains of Glu/Asp/Asn, and water molecules, but in contrast with the coordination of Ca2, some of these donor atoms are contributed by symmetry related molecules (S2 Fig), potentially suggesting less physiologically-relevant interactions. Nevertheless, the protein-metal interactions observed, especially for Ca2, are typical of the Ca^2+^ coordination schemes found in protein structures [48].

The two IgG-like domains of BP-2b_D2+D3_ contain two intra-molecular isopeptide bonds between residues Lys187 and Asn330 (in D2), and between Lys358 and Asn462 (in D3), as shown by the clear presence of continuous electron density between the side chains of the respective Lys and Asn residues (Fig 2). As previously observed for other backbone pilin subunits, the two isopeptide bonds are located within a highly hydrophobic region, and are stabilized by an Asp (residue 225) or a Glu (residue 423) of domains 2 and 3, respectively. These internal cross-links arise through autocatalytic, intramolecular reactions that occur spontaneously in the pilin subunits, aided by a neighboring Glu or Asp residue (D225 and E423 in BP-2b) [25, 49]. In addition, although both isopeptide bonds possess a trans peptide configuration, their arrangement with respect to the nearby catalytic Asp (for D2) or Glu (for D3) differs. Specifically, the isopeptide carbonyl O atom of Asn330 in D2 points away from the nearby Asp225, while the ε-amino group from Lys187 is positioned as such to be favorably contacted by the carboxylic group of the catalytic Asp225, with almost equally distant O atoms (3.3–3.6 Å) (Fig 2A). Instead, the carbonyl O atom of Asn462 in D3 points towards the carboxylic group of the catalytic Glu423, which in turns adopt a side-on position relative to the Asn462 side chain and thus forming only a single hydrogen bond (distance 2.7Å) (Fig 2B).

### Comparison with other Gram-positive pilin structures

**Fig 3:**
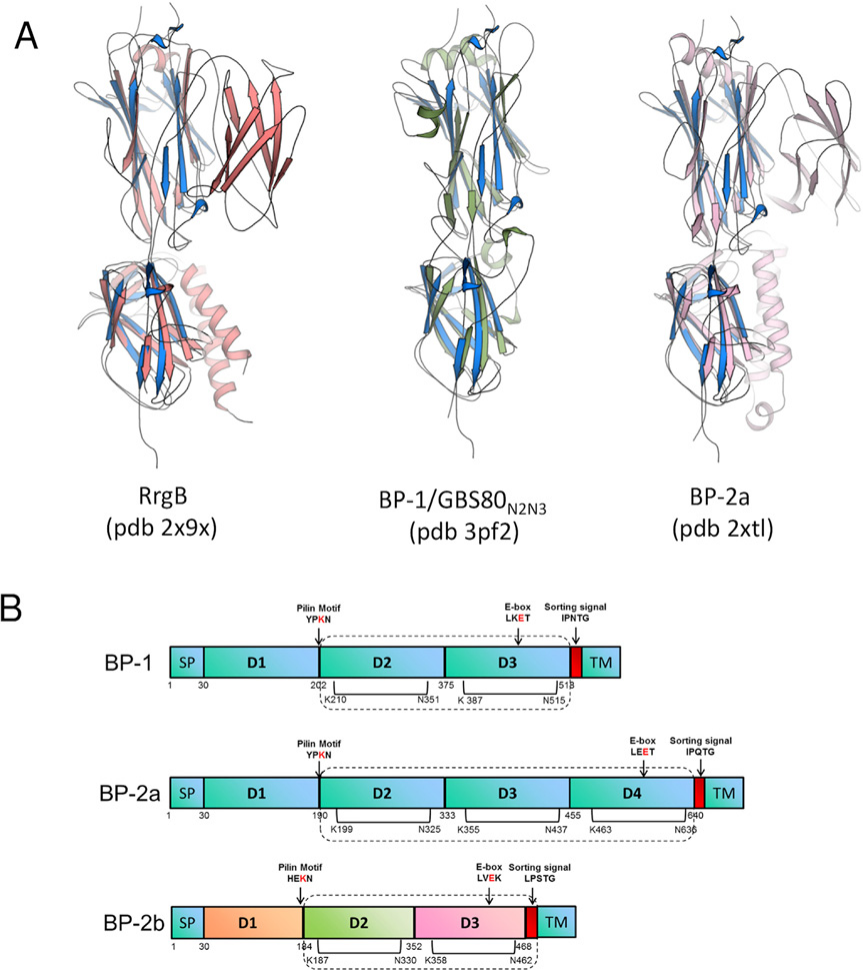
Structural comparisons of BP-2b_D2+D3_ with other pilin backbone proteins. (**A)** BP-2b (blue cartoon) is shown overlaid onto: the pilus backbone protein RrgB (pdb 2x9x, red cartoon, left), the major pilin protein GBS80 (pdb 3pf2, green cartoon, middle), and on the major pilin protein BP-2a (pdb 2xtl, pink cartoon, right). (**B)** Domain architecture of GBS backbone proteins from pilus 1 (BP-1), pilus 2a (BP-2a) and pilus 2b (BP-2b). The proteins are comprised of a signal peptide (SP) at the N-terminus and a C-terminal LPXTG-like motif (in red) linked to the transmembrane domain (TM). BP-1 and BP-2b contain three domains, while BP-2a four domains. The pilin motif involved in pilus polymerization is located near the D1–D2 domain linker while the E-box is located close to the sorting signal. Residues involved in isopeptide bonds are indicated by black bars. Domains present in the crystal structures are included into the box outlined with dashed lines.

A search of the protein data bank with the program DALI [50] revealed overall structural similarities with several cell wall surface anchor proteins, as well as with other pili, fimbrial, and collagen adhesion proteins. Although sequence identities were between 20 and 30%, the Z-scores obtained from DALI for the first 30 hits were between 13 and 17.5, indicating strong structural homologies. Among these first 30 hits, 11 could be uniquely identified (Table 2). Further structural comparisons were then performed by superpositions of GBS BP-2b_D2+D3_ onto the *S*. *pneumoniae* pilus backbone protein RrgB (pdb 2x9x) (rmsd 2.2Å for 209 common Cα atoms), the *S*. *agalactiae* major pilin proteins GBS80_N2N3_ (pdb 3pf2) (rmsd 2.5 Å for 205 common Cα atoms), and BP-2a (pdb 2xtl) (rmsd 2.3 Å for 212 common Cα atoms) (Fig 3A). The 3D structures of BP-2a and RrgB revealed an organization into four independently folded IgG-like domains (one CnaA and three CnaB domains) (Fig 3A and 3B). Interestingly, in these four-domain structures the third (D3) domain is positioned as a lateral insertion into the CnaA-type D2 domain with respect to the rest of the molecule (Fig 3A). Thus, the spatial arrangement of D2 and D4 domains overlaps the structural architecture of D2 and D3 domains of GBS BP-2b or BP-1 (Fig 3). These analyses further confirmed that the overall fold of the core of these proteins is very well conserved, although this cannot be readily predicted at the level of sequence.

**Table 2:** Multiple structural alignment of BP-2b protein with other known structures using the DALI server.

*PDB*	*Z score*	*rmsd*	*lali*	*nres*	*%ide*	*Description*	*Family*
2x9x	17.5	2.6	229	431	29	Pilus Backbone Pilin RrgB, *Streptococcus pneumoniae*	CELL WALL SURFACE ANCHOR FAMILY PROTEIN
3pf2	17.4	3.0	222	317	22	Major Pilin GBS80, *Streptococcus agalactiae*	CELL WALL SURFACE ANCHOR FAMILY PROTEIN
2xtl	17.2	2.7	230	449	25	Major Pilus Backbone Protein BP-2a, *Streptococcus agalactiae*	CELL WALL SURFACE ANCHOR FAMILY PROTEIN
4hsq	16.8	4.2	222	272	25	Major Pilin SpaD, *Corynebacterium diphtheriae*	PUTATIVE FIMBRIAL SUBUNIT
3rkp	16.5	3.8	221	350	23	Major Pilin Subunit BcpA, *Bacillus cereus*	COLLAGEN ADHESION PROTEIN
3htl	15.6	3.4	217	422	22	Major Pilin SpaA, *Corynebacterium diphtheriae*	PUTATIVE SURFACE-ANCHORED FIMBRIAL SUBUNIT
3uxf	15.6	3.8	225	449	24	Fimbrial Protein FimP, *Actonomyces oris*	FIMBRIAL SUBUNIT TYPE 1
3qdh	13.8	4.8	206	271	30	Fimbrial Adhesin FimA, *Actonomyces oris*	FIMBRIAL STRUCTURAL SUBUNIT
4igb	13.4	2.4	130	426	16	Adhesin Sgo0707, *Streptococcus gordonii*	LPXTG CELL WALL SURFACE PROTEIN
4oq1	13.2	3.9	129	331	21	Ancillary Pilin RrgC, *Streptococcus pneumoniae*	CELL WALL SURFACE ANCHOR FAMILY PROTEIN
3phs	13.0	2.5	99	239	28	Minor Pilin GBS52, *Streptococcus agalactiae*	Cell wall surface anchor family protein

Hits are ranked by Z-Score with best hits at the top of the table.

*PDB*: Protein Data Bank

*rmsd*: root-mean-square deviation of Cα atoms of superimposed proteins in Angstroms

*lali*: number of structurally equivalent positions

*nres*: number of structurally equivalent aligned residues

*%ide*: percentage of amino acid identity in aligned positions

### The LPXTG motif and the residues Lys175 and Glu423 are required for pilus 2b polymerization

To identify the specific residues and motives required for pilus 2b protein polymerization we used site-directed mutagenesis and complementation studies in GBS strains, as previously employed for the characterization of other GBS pili assembly [20, 51, 52].

**Fig 4:**
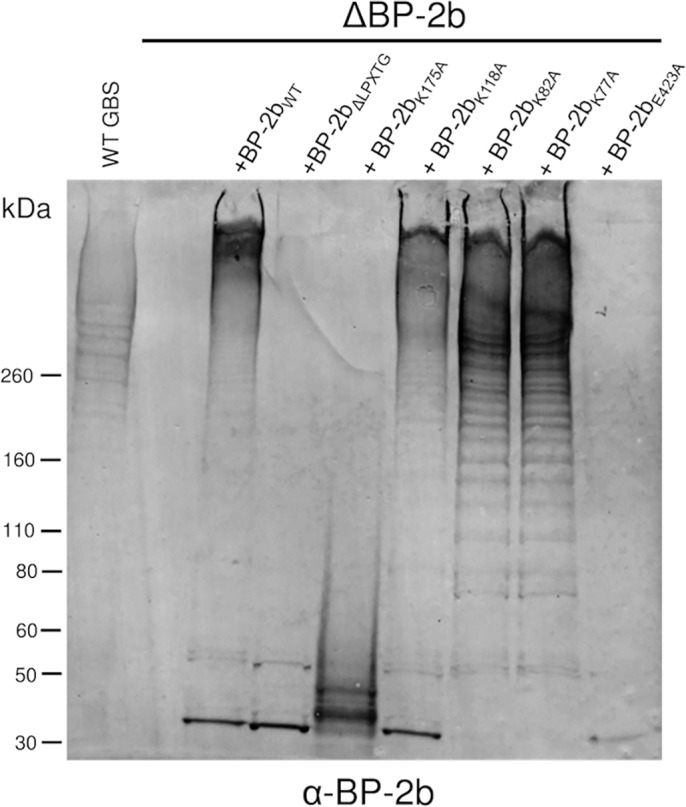
Lys 175, Glu 423 and the sorting motif LPSTG are involved in BP-2b polymerization in GBS. Immunoblot analysis of total protein extracts from GBS mutant strain lacking the pilus 2b backbone protein gene (Δ*BP-2b*) complemented with plasmids expressing the wild-type BP-2b protein (WT) or BP-2b mutants carrying a deletion of the C-terminal sorting signal (BP-2b_ΔLPXTG_), alanine substitutions of the putative pilin motif lysine (BP-2b_K175A_, BP-2b_K118A_ BP-2b_K82A_) or of the E-box E423 residue (BP-2b_E423A_). Nitrocellulose membrane was probed with a mouse antiserum raised against the recombinant BP-2b protein (α-BP–2b).

To confirm the key role of the sortase-recognition _GTE_LPSTG_GIG_ motif of the BP-2b protein in pilus assembly, this region was entirely deleted in the complementation plasmid pAM_BP-2b by site-directed mutagenesis. The new plasmid pAM-BP-2b_ΔLPXTG_ expressing the C-terminally truncated backbone subunit was used to complement the GBS mutant strain (Δ*BP-2b*) lacking the wild type gene for the pilus 2b backbone protein. The presence of covalently-linked pili on the GBS surface was detected by SDS-PAGE immunoblot analysis of cell-wall preparations through the identification of a ladder of high-molecular-weight (HMW) bands. Western blotting analysis, performed with total protein extracts from the complemented strain (ΔBP-2b/pAM-BP_ΔLPXTG_) and probed with a BP-2b specific antiserum, confirmed the expression of the protein only in the monomeric form, demonstrating that its polymerization into HMW structures was completely abolished (Fig 4).

To identify the specific lysine residue mediating the cross-linking between two monomeric subunits we performed a sequence analysis of the N-terminal domain D1 (which was not present in the crystallized fragment). Multiple alignments with the sequences of other backbone pilins (S3 Fig) showed a very low percentage of amino acid sequence similarity. BP-2b shared 22% and 24% sequence identity with the GBS backbone proteins from pilus 1 (BP-1) and pilus 2a (BP-2a), respectively. Moreover, the BP-2b primary sequence does not contain a “canonical” pilin motif, and four different lysine residues were identified in the N-terminal domain D1 as putative pilin motif candidates. To assess the role of each residue in pilus polymerization, each lysine in domain D1 was replaced individually by an alanine. By site-directed mutagenesis we generated four new complementation plasmids (pAM-BP-2b_K77A_, pAM-BP-2b_K82A_, pAM-BP-2b_K118A_, pAM-BP-2b_K175A_) and used them to transform the KO-strain ΔBP-2b. The complemented strains expressing mutated forms of BP-2b were analyzed for their ability to assemble HMW structures by immunoblotting analysis. We observed that the mutation of lysine 77, 82 and 118 into alanine did not affect pilus protein polymerization, while mutation of lysine 175 (K175A) led to the abrogation of pilus polymerization (Fig 4).

The identified pilin lysine 175, necessary for polymerization, is contained in the aminoacidic sequence V_TPN_A_T_IHEKN, completely different from the consensus of the canonical pilin motif (WxxxVxVYPKN).

In the BP-2b sequence, a canonical “LXET” E-box motif could not be identified; however, the BP-2b_D2+D3_ crystal structure showed that Glu423 (which is part of the motif LVEK) is favorably positioned towards the isopeptide bond between Lys358 and Asn462 in the D3 domain (Fig 2B), suggesting a possible role as for other E-box motives. To functionally characterize the role of this residue in pilus 2b polymerization, we constructed the plasmid pAM-BP-2b_E423A_ to complement the KO-strain ΔBP-2b. The expression of the backbone protein carrying the mutation E423A in the complemented strain abolished protein polymerization (Fig 4), demonstrating the key role of residue E423 in pilus 2b assembly, although present in a non-canonical E-box motif.

### The crystallized BP-2b_D2+D3_ fragment and the full length BP-2b have similar biochemical properties

**Fig 5:**
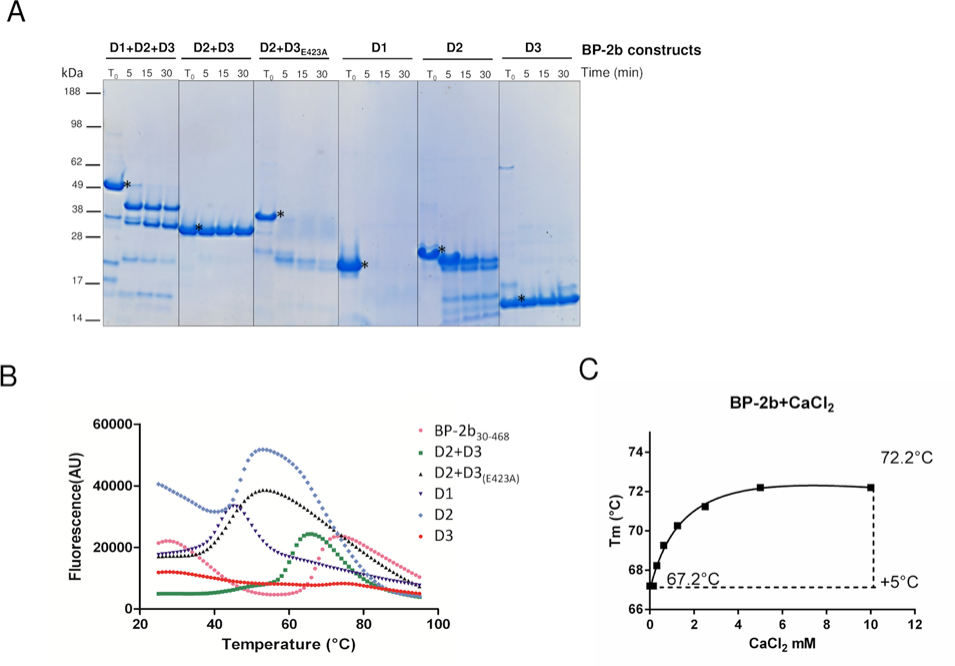
Biochemical characterization of different BP-2b constructs. **(A)** Time course of the trypsin-proteolysis reactions at 37°C of BP-2b full length and fragments, analyzed by SDS-PAGE. Different digestion patterns can be observed for the different constructs. Asterisks indicate the not-digested proteins. (**B)** Differential Scanning Fluorimetry (DSF) analysis of BP-2b proteins (D1+D2+D3, D2+D3 and single domains D1, D2, D3) in presence of Sypro orange showed different thermal stabilities. Graph shows the fluorescence intensity *vs*. the temperature for the unfolding different BP-2b constructs. (**C)** Correlation of BP-2b melting temperature with the concentration of Ca^2+^.

To investigate the differences in stability between the full length protein and single domains of BP-2b we performed limited proteolysis studies and thermal denaturation analysis. Firstly, we expressed in *E*. *coli*, and purified as recombinant His-tagged proteins different BP-2b constructs (the full length BP-2b_30-468_, BP-2b_D2+D3_, BP-2b_D2+D3(E423A)_, and the single D1, D2 and D3 domains) and probed the sensitivity of all generated proteins to limited proteolysis. Different Trypsin-BP-2b constructs digestion mixtures were prepared and tested at different time points (5, 15 and 30 min); the reactions were quenched by adding SDS sample buffer and analyzed by SDS-PAGE and Coomassie staining. The recombinant constructs of BP-2b differed in susceptibility to proteolysis (Fig 5A). The digestion pattern of the full length BP-2b showed the formation of multiple proteolytic products and the ∼30kDa band, corresponding to the BP_D2+D3_ fragment increased over the time. In contrast, the crystallized BP-2b_D2+D3_ construct was not affected by trypsin digestion, as well as the single domain D3. As already observed for other pilus proteins containing internal isopeptide bonds [49, 53], the mutant BP-2b_D2+D3(E423A)_, in which the glutamic acid E423 stabilizing the internal isopeptide bond in D3 domain is mutated, migrated on SDS-PAGE slightly slower than the native protein (lane T_0_ D2+D3 and lane T_0_ D2+D3_(E423A)_). The E423A mutant protein and the single D1 domain were completely degraded by trypsin after 5 minutes; while the D2 fragment was partially resistant to protease digestion. From these data, the crystallized BP-2b_D2+D3_ construct and the single D3 domain resulted to be the most resistant to proteolysis, probably due to intramolecular covalent (isopeptide) bond formation aiding in the formation of a more compact protein fold.

The thermal stability of recombinant BP-2b constructs (full length BP-2b, BP-2b_D2+D3_, and single D1, D2 and D3 domains) was assessed by differential scanning fluorimetry (DSF) using Sypro Orange as the external fluorescent probe, which binds to hydrophobic residues detecting their exposure during protein unfolding. The full length BP-2b and BP-2b_D2+D3_ proteins showed very low background fluorescence at 25°C and retained the intensity until around 60°C showing that the proteins are stable within this temperature range (Fig 5B). Heating above 60°C caused an increase in the fluorescent signal indicating protein unfolding upon heating. Our DSF analysis demonstrated that the recombinant full length BP-2b (containing D1, D2 and D3 domains) and BP-2b_D2+D3_ proteins exhibited the typical sigmoidal curve indicating that they contain domains that are well and similarly folded, with a Tm of 67°C and 61°C, respectively (Fig 5B).

D1 and D2 domains exhibited increased background fluorescence due to partially unfolded protein or presence of hydrophobic patches; however the melting process was still visible, showing that the isolated domains are less stable than the full-length or the BP-2b_D2+D3_ proteins, as their Tm was 40°C for D1 and 47.2°C for D2. Moreover, the Tm of D2+D3_(E423A)_ was 43°C, with a similar thermal denaturation curve of D1 and D2 domains, indicating that the glutamic acid residue (E423) in the putative E-box is important for protein folding and stability, as previously reported for the backbone protein of GBS pilus 2a (BP-2a) [20] and in accordance with our limited proteolysis results. For D3 domain, only a weak unfolding transition was observed, with a Tm of 71°C, suggesting that the domain D3 has a high thermal stability. Taken together, these data suggest a correlation between the thermal stability and susceptibility to proteolysis of the different recombinant BP-2b constructs, a trend that has been reported previously for other proteins [54].

Both the D2 and D3 domains of BP-2b presented bound Ca^2+^ ions in the crystal structure (S2 Fig). To test if BP-2b thermostability is enhanced by the addition of calcium ions, CaCl_2_ at different concentrations was added to the protein and the Tm was monitored by DSF. In the presence of Ca^2+^ ions, the *T*_*m*_ was observed to increase from 67.2 to 72.2°C, as the Ca^2+^ concentration in the solution was increased. The addition of >5 mM Ca^2+^ led to determination of the maximum value of the *T*_*m*_ (Fig 5C). This result indicated that the presence of a high concentration of Ca^2+^ contributes to increase the stability of BP-2b, a phenomenon that may also have promoted successful crystallization.

### BP-2b_D2+D3_ confers protection in mice

Finally, in order to study the immunological properties of BP_D2+D3_ compared with the full length protein and the single D1 domain, the purified recombinant proteins were independently used to immunize CD1 mice and the protection activity of each was tested in an active maternal mouse immunization/neonatal pup challenge model, as used previously for the identification of protein vaccine candidates for GBS [5, 6]. For the challenge, we used the GBS strain COH1 which expresses a high level of BP-2b protein on its surface. The BP_D2+D3_ construct conferred *in vivo* protection to a level comparable to that of the full length protein (Table 3), suggesting that protective epitopes in BP-2b are specifically concentrated in this portion of the protein, while the D1 domain appears dispensable for the protection in mice.

**Table 3:** Neonatal protection conferred by BP-2b domains vs the full length protein against GBS strain COH1 assessed by active maternal mouse immunization/neonatal pup challenge model.

Antigen	Protected/Treated	Protection (%)	Statistical significance (P value)*
BP-2b_D1_	10/49	15	0.0228
BP-2b_D2+D3_	31/46	65	P<0.0001
Full-length BP-2b	29/50	55	P<0.0001
PBS	4/67	0	

Groups of female mice received three doses (on days 1, 21, and 35) of either 20 μg antigen or buffer (PBS) combined in aluminium hydroxide formulations. Mice were then mated, and their offspring were challenged with a GBS dose calculated to induce death in 90% of the pups. Protection values were calculated as [(% dead in control—% dead in vaccine)/% dead in control] x100.

*P value, by Fisher’s exact test.

## Discussion

In this work we report the structural and biochemical characterization of the backbone protein of Group B *Streptococcus* (GBS) pilus 2b (BP-2b) that is one out of three pilus types identified in GBS by genome analysis [7, 55]. In the majority of GBS isolates pilus components are well exposed on the bacterial surface, immunogenic, and implicated at different stages of bacterial virulence and pathogenesis [5, 6, 10, 44, 56–63]. GBS pilus types 1 and 2a have been extensively studied in the last years, and the three-dimensional X-ray structures of their structural components (backbone and ancillary subunits) [8, 31–33] as well as of pilus-specific class C sortases [51, 52, 64, 65] are now available. By contrast, the pilus 2b remained so far uncharacterized, although the presence of the genomic pilus 2b locus is closely associated with the epidemiologically relevant lineage belonging to the highly virulent serotype III-sequence type (ST) 17, causing the majority of neonatal invasive diseases [12, 66, 67].

The three-dimensional crystal structure of a proteolysis-resistant fragment of BP-2b reveals an architecture that is typical of the pilin subunits in Gram-positive bacteria. Despite high variations in size, number of domains and low sequence homologies, the available crystal structures show that the pilin subunits are generally made of two types of IgG-like domains, CnaA and CnaB, with similar folding and structural organization, in which each modular domain is stabilized by intramolecular auto-catalytically formed isopeptide bonds between the side chains of Lys and Asn residues [16]. The BP-2b structure shows a three-domain organization with a CnaA-type central domain (D2), and a C-terminal CnaB-type domain (D3) that carries the LPxTG-like sorting motif, closely resembling the structural architecture of the backbone protein of GBS pilus 1 (BP-1, GBS80) [33], the shaft pilins SpaA and SpaD from *Corynebacterium diphtheriae* [21, 22] and FimA and FimP from *Actinomyces oris* [23, 24]. Interestingly, the overall fold of BP-2b remains highly conserved when compare with all other pilin structures available (Fig 3A), independently from the number of Ig-G-like domains present in the structure and levels of sequence conservation.

Successful crystallization was achieved with the BP-2b construct encompassing domains D2 and D3 and lacking of the N-terminal domain D1. Many crystal structures of other pilins, including the major subunit of GBS pilus 1 [33] and pilus 2a [8] as well as *A*. *oris* FimA [23], *B*. *cereus* BcpA [34], and *S*. *pneumoniae* RrgB [26] are missing of the N-terminal domain, apparently resulting particularly sensitive to proteases. This is likely due to an intrinsic high flexibility, as revealed also in the structures of FimP from *A*.*oris* [24] and SpaA and SpaD from *C*. *diphtheriae* [21, 22] that have been crystallized in the full length form. We have identified the lysine residue involved in the intermolecular linkages by site-directed mutagenesis and complementation studies. This residue (Lys175) is present in a “non-canonical” pilin motif and located at the end of the N-terminal domain D1. It is tempting to speculate that Lys175 is located within D1 in a similar fashion to the pilin motif of FimP [24] or SpaD [22], where is positioned in a groove at the interface between the first and second domain and surrounded by conserved residues that may function as a docking site for the sortase-pilin complex.

The protease- and thermal susceptibility of D1 domain of BP-2b has been confirmed by our analysis of limited proteolysis and thermal denaturation. By contrast, the high resistance of D2–D3 domains to proteolytic and thermal denaturation, already known for other pilins [68], is most likely due to the presence in each domain of Lys-Asn isopeptide bonds located in a predominantly hydrophobic pocket and nearby bond-catalyzing aspartyl or glutamyl residues. These covalent intra-domain linkages are a widespread feature among Gram-positive pilins, and are known to increase the stability of the surface proteins by conferring resistance to proteases and mechanical forces/stresses, especially when bacteria engaging host cells during bacterial colonization [68]. The BP-2b_D2+D3_ structure shows that the conserved E-box residue (E423) is favorably positioned towards the isopeptide bond between Lys358 and Asn462 in the D3 domain, contributing to stabilize it and to confer a proper folding to the protein. Indeed, its substitution with an alanine residue completely abolishes the pilus polymerization and makes the mutated protein highly sensitive to proteolysis, in agreement with the phenotype already observed for the E-box-mutant of the backbone protein of pilus 2a [20].

The N-terminal flexibility of D1 domains as well as the presence of isopeptide bonds is expected to have implications for pilus assembly. This flexibility is assumed to facilitate docking of the sortase enzymes and allow the formation of the intermolecular linkage between the C-terminal sorting motif of one molecule with the N-domain of the next during pilus polymerization. The subsequent formation of an internal isopeptide bond in D1 domain may contribute to stabilize the growing polymerized pilus structure [16]. The relationship between internal isopeptide-bond formation and pilus assembly has been clearly demonstrated by structural studies of the pneumococcal major pilin RrgB. The crystal structure of the full length RrgB protein carrying also the C-terminal sorting signal revealed a pseudo-pilus polymer of successive molecules, with the sorting motif of one molecule docked on to the N-domain of the next. Interestingly, the isopeptide bond in the D1 domain was formed in this structure [28]. Conversely, in a previous study, the full-length monomeric RrgB structure showed a flexible N-domain in which the internal isopeptide bond was not formed [29]. This is also in agreement with mass-spectral (MS) data obtained for pili of *B*. *cereus* [34] and *C diphtheriae* [22]. In the N-terminal D1 domain of the major subunit *B*. *cereus* BcpA the presence of an isopeptide bond has been demonstrated in native pili only after pilin subunits have been incorporated into the polymerized structure [34]. In *C*. *diphtheriae* pili, the crystal structure of the full-length major subunit SpaD revealed the presence of an isopeptide bond in the N-terminal D1 domain. However, MS analysis showed that this linkage formed slowly, probably because of its spatial location close to the lysine involved in the sortase-mediated intermolecular linkage with the next pilin during pilus assembly [22].

In addition to the intramolecular isopeptide bonds in each domain the BP-2b structure present another stabilizing feature. In both D2 and D3 domains, a metal binding site is formed. The coordination environment and average metal-ligand bond length (2.4 Å) are indicative of a Ca^2+^ ion, presumably derived from the crystallization conditions (solution containing 0.2 M calcium acetate) [48]. Protein thermal stability was measured in the presence of calcium ions by DSF, showing that the addition of calcium chloride significantly increased the thermal stability of the protein. Calcium-binding sites appear to be a common feature of other pilin proteins, as *C*. *diphtheriae* SpaA and SpaD [21, 22], *A*. *oris* FimA [23] and FimP [24] and GBS BP-1 [33]. Although their location is not conserved and their putative role has not been studied, it is commonly thought that these sites contribute to protein stability.

Our study suggests that the crystallized BP-2b_D2+D3_ fragment and the BP-2b full length protein have similar biochemical and immunological properties. The BP-2b_D2+D3_ construct, lacking the N-terminal protease-sensitive domain, is equivalent in terms of folding to the full length protein, strengthening the idea that both D2 and D3 domains are necessary for the overall conformational stability of the protein. Interestingly, the BP-2b_D2+D3_ fragment retains also the ability to confer protection against bacterial infection in a well-established animal model whereas the N-terminal D1 domain contains nonfunctional epitopes which are dispensable for protection. In GBS all three pili carry subunits able to elicit protective immunity in mice and the combination of pilus components can provide broad protection against circulating GBS strains [6]. Thus, the identification of the immunodominant region of BP-2b by combining structural, biochemical and functional information provides additional insights into the native molecular architecture of protective determinants and offers opportunities for therapeutic intervention against pathogenic bacteria, and for a structure-based design of new vaccine formulation.

## Supporting Information

S1 Fig Analysis of the protease-resistant BP-2b fragment. **A)** Crystals of the recombinant BP-2b_D2+D3_ protein, grown from condition A7 of a PEG/ION screen (Hampton Research), containing 0.2 M calcium acetate and 20% (w/v) polyethylene glycol 3,350 in approximately three weeks. **B)** SDS-PAGE of the recombinant BP-2b_30-468_ protein, expressed in, and purified from *E*. *coli* and stored at 4°C or at -20°C for approximately six months. **C)** SDS-PAGE of the purified recombinant protease-resistant BP-2b_185-468_ fragment after immobilized-metal affinity chromatography (IMAC) and size-exclusion chromatography (SEC). Gels were Coomassie Blue G-250 stained. **D)** Gel filtration chromatogram of the BP-2b_185-468_ fragment recorded at 280 nm wavelength. To estimate BP-2b_D2+D3_ size, a high molecular weight standard kit (Biorad, 151–1901) containing Thyroglobulin (670 kDa) elution volume (Ev) 9.43 ml, γ-globulin (158 kDa) Ev 12.76 ml, Ovalbumin (44 kDa) Ev 15.38 ml, Myoglobin (17,000 Da) Ev 17.8 ml, was run in the same column (semi-analytical superdex 200 10/300 GL) and with the same buffer conditions.
(TIF)

S2 Fig Calcium ions bound to BP-2b. The structure of BP-2b_185-468_ is shown in the middle and depicted as in Fig 1, and zoom into the local environment of each of the three calcium ions modelled in the final structure are presented in boxes. Blue and orange depict domains D2 and D3 of BP-2b, respectively, while grey sticks and spheres show atoms from symmetry-related molecules that are involved in the coordination of calcium ions 1 and 3.
(TIF)

S3 Fig Sequence comparison of BP-2a with other backbone pilins. Multiple sequence alignment performed by using Mafft and ESPrit of BP-2b primary sequence with *S*. *pneumoniae* RrgB, GBS BP-1 and BP-2a, *C*. *diphtheriae* SpaA and SpaD, *Bacillus cereus* BcpA and *A*. *naeslundii*. FimA and FimP). Identical residues are shown with a red background, whereas similar residues are shown in red and highlighted with blue boxes.
(PDF)
