# Supplementary figures and images for: Structure and Assembly of Group B Streptococcus Pilus 2b Backbone Protein
Source: PLoS One. 2015 May 5;10(5):e0125875. doi: 10.1371/journal.pone.0125875 (PMC4420484; doi:10.1371/journal.pone.0125875)

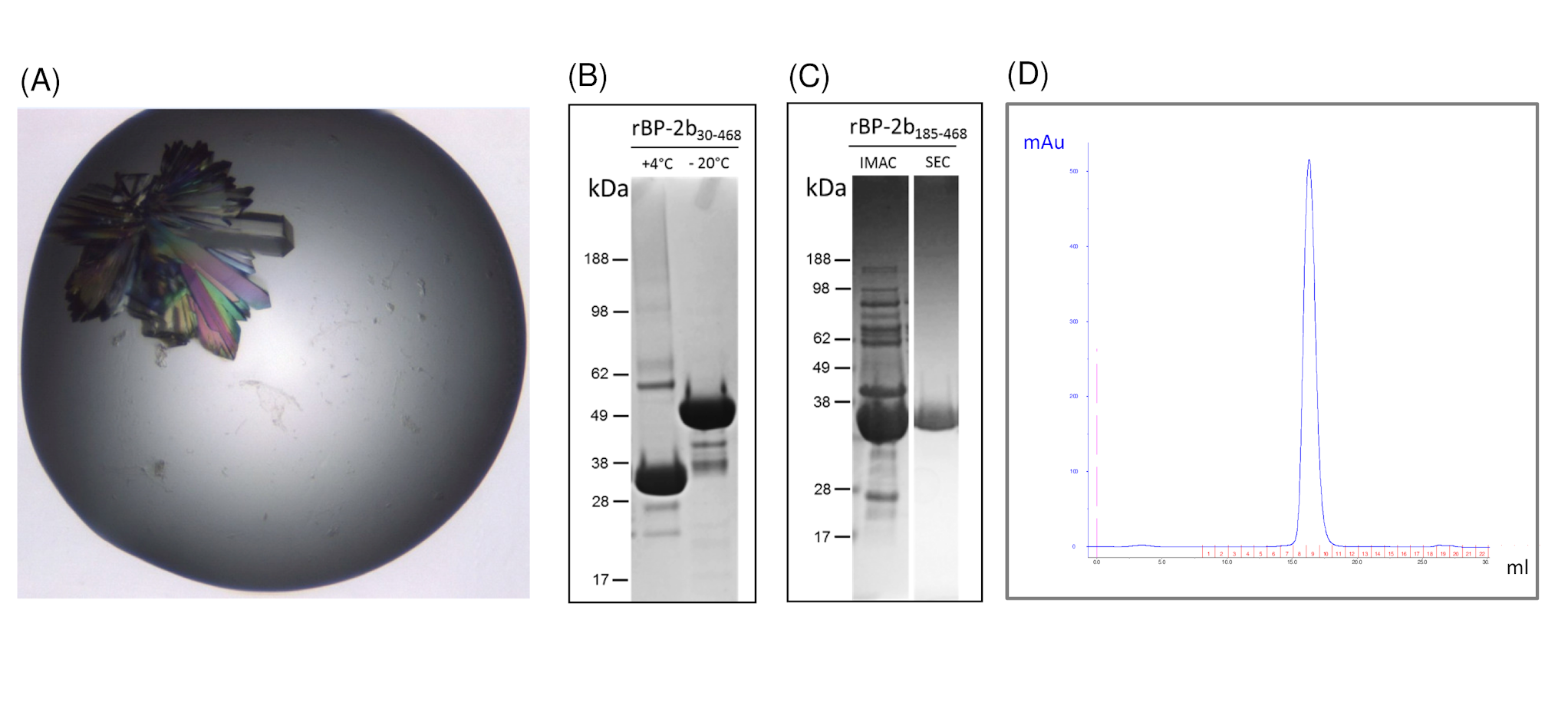

Supplement: S1 Fig — A) Crystals of the recombinant BP-2bD2+D3 protein, grown from condition A7 of a PEG/ION screen (Hampton Research), containing 0.2 M calcium acetate and 20% (w/v) polyethylene glycol 3,350 in approximately three weeks. B) SDS-PAGE of the recombinant BP-2b30-468 protein, expressed in, and purified from E. coli and stored at 4°C or at -20°C for approximately six months. C) SDS-PAGE of the purified recombinant protease-resistant BP-2b185-468 fragment after immobilized-metal affinity chromatography (IMAC) and size-exclusion chromatography (SEC). Gels were Coomassie Blue G-250 stained. D) Gel filtration chromatogram of the BP-2b185-468 fragment recorded at 280 nm wavelength. To estimate BP-2bD2+D3 size, a high molecular weight standard kit (Biorad, 151–1901) containing Thyroglobulin (670 kDa) elution volume (Ev) 9.43 ml, γ-globulin (158 kDa) Ev 12.76 ml, Ovalbumin (44 kDa) Ev 15.38 ml, Myoglobin (17,000 Da) Ev 17.8 ml, was run in the same column (semi-analytical superdex 200 10/300 GL) and with the same buffer conditions. (TIF) [file pone.0125875.s001.tif]

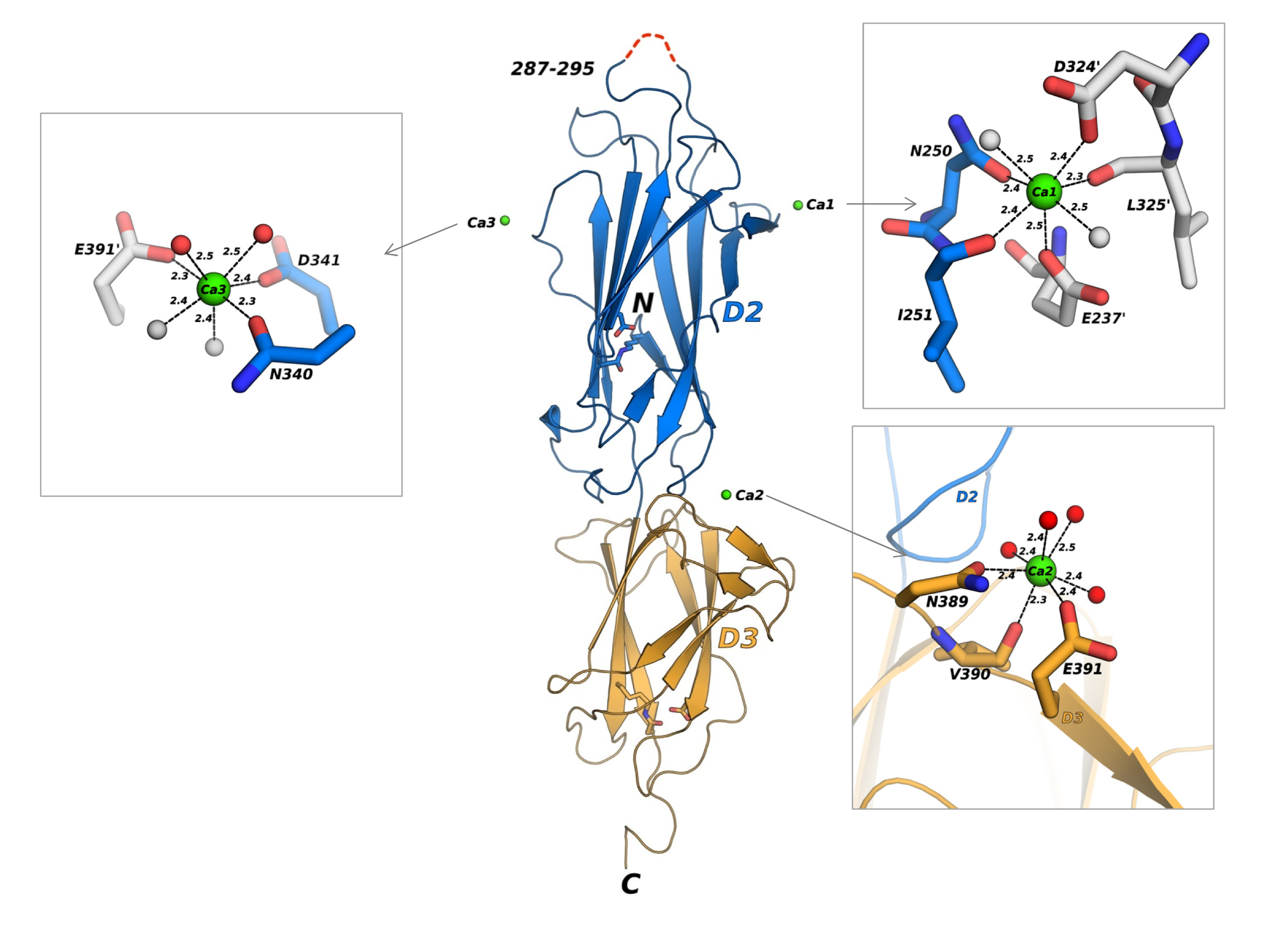

Supplement: S2 Fig — The structure of BP-2b185-468 is shown in the middle and depicted as in Fig 1, and zoom into the local environment of each of the three calcium ions modelled in the final structure are presented in boxes. Blue and orange depict domains D2 and D3 of BP-2b, respectively, while grey sticks and spheres show atoms from symmetry-related molecules that are involved in the coordination of calcium ions 1 and 3. (TIF) [file pone.0125875.s002.tif]
