# Supplementary material for: Structure and Assembly of Group B Streptococcus Pilus 2b Backbone Protein
Source: PLoS One. 2015 May 5;10(5):e0125875. doi: 10.1371/journal.pone.0125875 (PMC4420484; doi:10.1371/journal.pone.0125875)
Supplement: S3 Fig — Multiple sequence alignment performed by using Mafft and ESPrit of BP-2b primary sequence with S. pneumoniae RrgB, GBS BP-1 and BP-2a, C. diphtheriae SpaA and SpaD, Bacillus cereus BcpA and A. naeslundii. FimA and FimP). Identical residues are shown with a red background, whereas similar residues are shown in red and highlighted with blue boxes. (PDF) [file pone.0125875.s003.pdf]

```
              1              10        20        30
GBS_BP-2b     M.......KKKMIQSLLVASLAFGMAVSPVTPIAFAA....................ETG
Pneumo_RrgB   M.....KSINKFLTMLAALLLTASSLFSAATVFAAGT....................TTT
GBS_BP-1      M.....KLSKKLLFSAAVLTMVAGSTVEPVAQFATGMSIVRAAEVS.......QERPAKT
GBS_BP-2a     M.....KKINKYFAVFSALLLTVTSLFSVAPVFAE...................EAKTTD
Dipht_SpaD    MKKNSLTIRSVTFAAVAGLSLGISA.PGAVAIAEEGTQVQQTQRAN.......IDFERKG
Cereus_BcpA   M........KKIFSVLLVLFLTFST.WSSVLVKA....................DSPSKG
Dipht_SpaA    M.....TARGCRLATLATTLTFFVSNVTLASVFAEESQYHAVQLGESPLSVDGLSIPERT
Actinom_FimP  M...HSLNTRRGLGLAAAMTLAAGALVAPTGAAAPAD....PNGST.......IDPDAAT
Actinom_FimA  MKHNASTLGRRAAAAAGVLTLAFLG.LAPSAVATET.....PNYGN.......IKTDATG

                   40         50        60                70        80
GBS_BP-2b     TITVQDTQK.GATYKAYKVFDAEIDNANV...SDSNKD.....GASYLIPQGKEAEYKAS
Pneumo_RrgB   SVTVHKLLATDGDMDKI...ANELETGNY.AGNKVGVL.....PANAKEIAGVMFVWTNT
GBS_BP-1      TVNIYKLQ..ADSYKSEITSNGGIENKDGEVISNYAKL.....GDNVKGLQGVQFKRYKV
GBS_BP-2a     TVTLHKIVMPRTAFDGFTAGTKGKDNTDY.VGKQIEDLKTYFGSGEAKEIAGAYFAFKNE
Dipht_SpaD    SLTLHK....KKGAESEKRATGKEMDDVA.................GEPLNGVTFKITKL
Cereus_BcpA   TLTIHKYEQEKDGAQGLEGDGSANQEVPK................DVKPLKGVTFEVKRV
Dipht_SpaA    SIAVHALM....GLPTGQPANGTKLDSIG.................LPKVDGMSFTLYRV
Actinom_FimP  TLTVHKCE..QTDTNGVKEGTGNEDPQAE.................CKPVSDVEFTITKL
Actinom_FimA  SLAIHKHL..NGGGKDIGNPTGTPQNADS................KGTPVQGVVFTAYPI

                  90       100            110       120       130
GBS_BP-2b     TDFNSLFTTTTNGGRTYVTKK.....DTASANEIATWAKSISANTTPVSTVTESNNDGTE
Pneumo_RrgB   .NNE...IIDENGQTLGVNID....PQTFKLSGAMPATAM........KKLTEAEGAK..
GBS_BP-1      .KTD...ISVDE.LKKLTTVE....AADAKVGTILEEGVS.......LPQKTNAQGLVVD
GBS_BP-2a     AGTK...YITENGEEV..........DTLDTTDAKGCAVL........KGLTTDNGFK..
Dipht_SpaD    .NFD...LQNGD.WAKFPKTA.......ADAKGHETSTTK........EVETSGNGTA..
Cereus_BcpA   ASFE...KISNDGKIVKEDVK...........PVMGATPN........QVVTDDNGQA..
Dipht_SpaA    NEID...LTTQAGWDAASKIK....LEELYTNGHPTDKVT......KVATKKTEGGVA..
Actinom_FimP  .NVD...LTTYDGWKTLADLK....GDVVKAGALKSTTVQ........KITTGANGLA..
Actinom_FimA  TDIN...LKDPAGWDTISDLSKAGVPDSACTNPAAPTLGAHKFGTPKVSPATNDEGLA..

            140         150               160
GBS_BP-2b     VINV..SQYGYYY.VSST.......VNNGAVIMVTSVTP....................N
Pneumo_RrgB   .FNTANLPAAKYK.IYEIHSLSTYVGEDGATLTGSKAVPIEIELPLND.........VVD
GBS_BP-1      ALDS..KSNVRYLYVEDL.......KNSPSNITKAYAVPFVLELPVANSTGTG...FLSE
GBS_BP-2a     .FNTSKL.TGTYQ.IVELKEKSTY.NNDGSILADSKAVPVKITLPLVNDNG.....VVKD
Dipht_SpaD    VFDN..LDLGIYL.VEET........KAPDGIVT..GAPFIVSIPMVNEASDA...WNYN
Cereus_BcpA   VLKD..LPLGRYE.VKEV........AGPPHVNLN.PNTYTVDIPLTNKEGKV...LNYD
Dipht_SpaA    KFDN..LTPALYLVVQEL........NGAEAVVR..SQPFLVAAPQTNPTGDG...WLQD
Actinom_FimP  SFTDAQTEVGAYL.VSET........RTPDKVIP..AEDFVVTLPMTNPQDTA..KWNYN
Actinom_FimA  TITE..MPVQAYL.VCET........TTPGNIVQK.AKPFVVTVPHPNTAAGADGQWIYD

            170       180         190
GBS_BP-2b     ATIHEKNTDAT..WGDGGGKTVDQK...................................
Pneumo_RrgB   AHVYPKNTEAK.PKID...KDFKGKANPDT..............................
GBS_BP-1      INIYPKNVVTDEPKTD...KDVKKLGQDDA..............................
GBS_BP-2a     AHVYPKNTETK.PQVD...KNFADKELDYA..............................
Dipht_SpaD    VVAYPKNTETK...TE...KTVKDADQN................................
Cereus_BcpA   VHMYPKNEIKR.GAVDLIKTGVNEKALAGAVFSLFKKDGTEVKKELATDANGHIRVQGLE
Dipht_SpaA    VHVYPKHQALS........EPVKTAVDPDA..............................
Actinom_FimP  VHVYPKNTLSG...VD...KQVTDKPAP................................
Actinom_FimA  VHVYPKNEAIS...VE...KSIQEQKLN................................

                                                       200
GBS_BP-2b     ....................................TYSVGDTVK...............
Pneumo_RrgB   ...........................PRVDKDTPVNHQVGDVVE...............
GBS_BP-1      ....................................GYTIGEEFK...............
GBS_BP-2a     ...........................NNKKDKGTVSASVGDVKK...............
Dipht_SpaD    .......................................IQDAYT...............
Cereus_BcpA   YGEYYFQETKAPKGYVIDPTKREFFVKNSGTINEDGTITSGTVVKIEVKNNEEPTIDKKI
Dipht_SpaA    .................................TQPGFSVGENVK...............
Actinom_FimP  ......................................GSGRDIT...............
Actinom_FimA  ....................................GYGVGSLIK...............
```

```
                               210       220       230
GBS_BP-2b     ................YTITYKNAV.NYHGTEKVYQYVIKDTMPS.......ASVVDLNE
Pneumo_RrgB   ................YEIVTKIPA.....LANYATANWSDRMTEGLAFNKGTVKV....
GBS_BP-1      ................WFLKSTIPA....NLGDYEKFEITDKFADGLTYKS.VGKI....
GBS_BP-2a     ................YHVGTKILK.....GSDYKKLIWTDSMTKGLTFNN.DIAV....
Dipht_SpaD    ................YTIKADAPT..WGKDKKLTAFRFEDELDKRLDFQK.VTEV....
Cereus_BcpA   NGKLEALPINPLTNYNYDIKTLIPE....DIKEYKKYVVTDTLDNRLVIQG.KPIV....
Dipht_SpaA    ................YRVATKIPE..IASNTKFEGFTVADKLPAELGKPD.TNKITV..
Actinom_FimP  ................YTITTSIPKVDYPGGARIKRYEVVDRLDKRIKKEA.LTPVVKIV
Actinom_FimA  ................FPVSSTAPT..LDAKSFYKYFQLRDTLDDRLTAVT.ATEV....

              240       250       260       270       280
GBS_BP-2b     GSYEVTITDGSGNITTLT.QGSEKATGKYNLLEENNNFT........ITIPWAATNTPTG
Pneumo_RrgB   .TVDDVALEAGD.YALTE....VATGFDLKLTDAGLAKV................NDQNA
GBS_BP-1      .KIGSKTLNRDEHYTIDEPTVDNQNTLKITFKPEKFKEIAELLKGMTLVKNQDALDKATA
GBS_BP-2a     .TLDGATLDATN.YKLVA....DDQGFRLVLTDKGLEAV................AKAAK
Dipht_SpaD    .KAGDTVLGTSD.YTVND.PATDGNKLVVTLTDEGLKKV.....................
Cereus_BcpA   .KIDGAEVNANV.VEVAI....EGQKVTATVKD..FTKL.....................
Dipht_SpaA    .TLGGKPINSTD.VSVQTYQVGDRTVLSVQLAGATLQSL.....................
Actinom_FimP  GQNEVTLAETTD.YTLITAEGKDHNWATIQLTEEGRRKA................SEARY
Actinom_FimA  .SLEGTTLDPTD.YKVDT....KGQTVTVTFTAEGLKKI.....................

             290       300       310
GBS_BP-2b     NTQN..GANDDFFYKGINTITVTYTGVL................................
Pneumo_RrgB   EKTV..KITYSATLNDKAIVEVPESNDVTFNYGNNPDHGNTPKPNKPNENGDLTLTKTWV
GBS_BP-1      NTDD..AAFLEIPVAST...........................................
GBS_BP-2a     TKDVEIKITYSATLNGSAVVEVLETNDVKLDYGNNPTIENEPKEGIPVDK.KITVNKTWA
Dipht_SpaD    ..KS..GDKMSLTFEVK...........................................
Cereus_BcpA   ..DG..KKEFHLQIKSQ...........................................
Dipht_SpaA    DQHK..DQELVVEFEAP...........................................
Actinom_FimP  NGNG..ETKLQVTLNAK...........................................
Actinom_FimA  KAAP..GKKVSAVFQGK...........................................

GBS_BP-2b     ............................................................
Pneumo_RrgB   DATGAPIPAGAEATFDLVNAQTGKVVQTVTLTTDKNTV......TVNGLDKNTEYKFVER
GBS_BP-1      ............................................................
GBS_BP-2a     .VDGNEVNKADETVDAVFTLQVKDGDKWVNVDSAKATAATSFKHTFENLDNAKTYRVIER
Dipht_SpaD    ............................................................
Cereus_BcpA   ............................................................
Dipht_SpaA    ............................................................
Actinom_FimP  ............................................................
Actinom_FimA  ............................................................

                  320       330                 340                350
GBS_BP-2b     .KSGAKPGSADLPENTNIAT..........INPNT....SNDDPG........QKVTVRD
Pneumo_RrgB   SIKGYSADYQEITTAGEIAV..........KNWKD....ENPKPLDP....TEPKVVT.Y
GBS_BP-1      .INEKAVLGKAIENTFELQY..........DHTPD....KADNPK.PSNPPRKPEVHT.G
GBS_BP-2a     .VSGYAPEYVSFVNGVVTIK..........NNKDS....NEPTPINP....SEPKVVT.Y
Dipht_SpaD    .RKEV.GNTTELKNRADVIF..........NNPNT....DKEVKN......KTNEVVTYH
Cereus_BcpA   .VKEGVPSGSEILNTAKIHF..........TNKNDVIGEKESKPV.........VVIPTT
Dipht_SpaA    .VTKQ.PENGQLDNQAWVLP..........SNPTA.QWDPEESGDAALRGMPSSRVSSKF
Actinom_FimP  .FDAAVNLEGDLSNTAGLIPNDSPNFTWDPNNPGT....TTDIPGIP.....TTPVLSKY
Actinom_FimA  .VTEA..RNGAITNRAQVIS.....DTVYAEQPPT....PEEPPANPENPPTSNEVTSRW

                     360       370       380
GBS_BP-2b     GQITIKKI.DGST....KASLQGAIFVLKN..ATGQFLNFND..................
Pneumo_RrgB   GKKFVKVN.DKDN......RLAGAEFVIANADNAGQYLARKADKVSQEEKQLVVTTKDAL
GBS_BP-1      GKRFVKK..DSTE....TQTLGGAEFDLLA..SDGTAV......................
GBS_BP-2a     GRKFVKTNKDGKE......RLAGATFLVKK...DGKYLARKSGVATDAEKAAVDSTKSAL
Dipht_SpaD    GKLKVVKK.DGKEAGK...VLKGAEFELYQ..CTSAAV......................
Cereus_BcpA   GIIELTKI.DSAN....KNKLKGAEFVLKD..NNGKIV......................
Dipht_SpaA    GQITIEKSFDGNTPGADRT....ATFQLHRCEADGSLV......................
Actinom_FimP  GKVVLTKT..GTDDLADKTKYNGAQFQVYE..CTKTASGATL..................
Actinom_FimA  GDLLIKKV.DNHQQGQDKAGLQGAQFQLYK..AKNAYAGTCT..................
```

```
                                               390        400
GBS_BP-2b     ................................TNNVEWGTEANATEYTTGA.........
Pneumo_RrgB   DRAVAAYNALTAQQQTQQEKEK....VDKAQAAYNAAVIAANNAFEWVADK.DNENVVKL
GBS_BP-1      ................................KWTDALIKANTNKNYIAGEAVTGQPIKL
GBS_BP-2a     DAAVKAYNDLTKEKQEGQDGKSALATVSEKQKAYNDAFVKANYSYEWVEDK.NAKNVVKL
Dipht_SpaD    .................................LGKGPLTVDGVKKWTTGD.........
Cereus_BcpA   ......................................VVAGKEVTGVSDE.........
Dipht_SpaA    .................................KSDPPISLDGKQEFVTGQ.........
Actinom_FimP  ..........................RDSDPSTQTVDPLTIGGEKTFTTAG.........
Actinom_FimA  ...............................KDKEGDPIAINGETTLTTDA.........

                     410                           420       430       440
GBS_BP-2b     ....DGIITITGLKEG....................TYYLVEKKAPLGYNLLDNSQKVI.
Pneumo_RrgB   VSDAQGRFEITGLLAG....................TYYLEETKQPAGYALLTSRQKFEV
GBS_BP-1      KSHTDGTFEIKGLAYAVDANAEGTAV..........TYKLKETKAPEGYVIPDKEIEFTV
GBS_BP-2a     ISNDKGQFEITGLTEG....................QYSLEETQAPTGYAKLSGDVSFNV
Dipht_SpaD    ....DGTFTIDGLHVTDFEDGKEAAPATK.......KFCLKETKAPAGYALPDPNV.TEI
Cereus_BcpA   ....NGVIKWSNIPYG....................DYQIFETKAPT.YTKEDG......
Dipht_SpaA    ....DGKAVLSGIHLGTLQLESNVMKYTDAWAGKGTEFCLVETATASGYELLPKPVIVKL
Actinom_FimP  ....QGTVEINYLRANDYVNGAKKDQLTDED.....YYCLVETKAPEGYNLQADPLPFRV
Actinom_FimA  ....QGAINVKGLFISDSIDGANRDNQKDATAR...CYVLVETKAPAGYVLPAGDG....

                            450       460         470       480
GBS_BP-2b     .......LGDGATDTTNSDNLLVNPTVENN..KGTELPSTGGIGTTIFYIIGAI..LVIG
Pneumo_RrgB   TATSY..SATGQGIEYTAGSGKDDATKVVN..KKITIPQTGGIGTIIFAVAGAV..IMGI
GBS_BP-1      SQTSY..NTKPTDITVDSADATPD.TIKNN..KRPSIPNTGGIGTAIFVAIGAA..VMAF
GBS_BP-2a     NATSYS.KGSAQDIEYTQGSKTKDAQQVIN..KKVTIPQTGGIGTIFFTIIGLS..IMLG
Dipht_SpaD    EFTRAK.ISEKDKFEGDDEVTLVS.EIKNIKQGTPKLPMTGGAGVGILAAIGAA..IVAA
Cereus_BcpA   TKTSYQLLKDPIDVKISENNQTVKLTIENNK.SGWILPVTGGIGTTLFTVIGLT..LMLT
Dipht_SpaA    E......ANESTNVLVEQKVKIDN..KKKN..AGFELPLTGGSGRIAITI.GIVGLLVAL
Actinom_FimP  .......LAEKAEKKAATEVTVTD..IPKN..AGFRLPLTGANGVIFLTIAGAL..LVAG
Actinom_FimA  .......AVTPVKIEVGA.VTTDNVTIENTKQSVPGLPLTGANGMLILTASGASLLMIAV

            490          500
GBS_BP-2b     AGIVLVA...RRRLR...S
Pneumo_RrgB   AVYAYVK...NNKDEDQLA
GBS_BP-1      AVKGMKR...RTKDN....
GBS_BP-2a     AVVIMKR...RQSEE...V
Dipht_SpaD    GAWFARR...GAKN.....
Cereus_BcpA   AAFVFFR...KKFAR...N
Dipht_SpaA    ASYVLSR...RKDNR....
Actinom_FimP  GAVVAYA...NKRRHVAKH
Actinom_FimA  GSVLVARYRERKQNANLAL
```
